# Supplementary material for: Staphylococcus epidermidis Cicaria, a Novel Strain Derived from the Human Microbiome, and Its Efficacy as a Treatment for Hair Loss
Source: Molecules. 2022 Aug 12;27(16):5136. doi: 10.3390/molecules27165136 (PMC9414784; doi:10.3390/molecules27165136)
Supplement: Supplementary file 1 [file molecules-27-05136-s001.zip › molecules-1736817-supplementary.pdf]

Supplementary Material

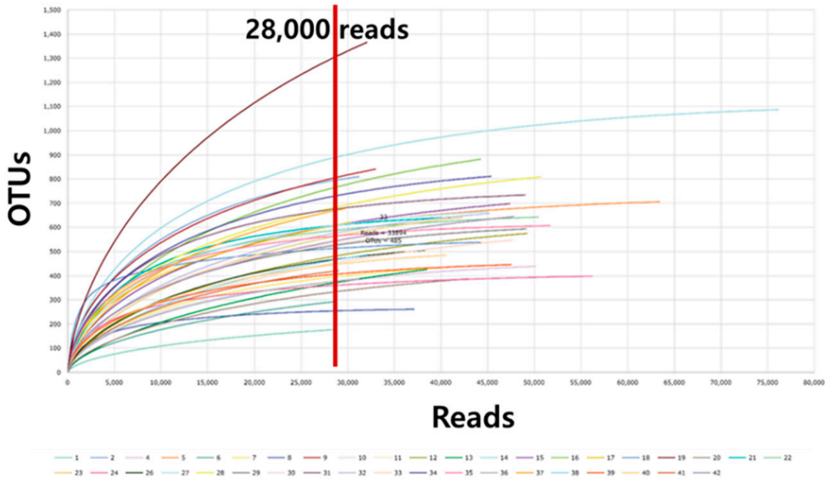

Figure S1. Sequencing reads of the scalp samples.

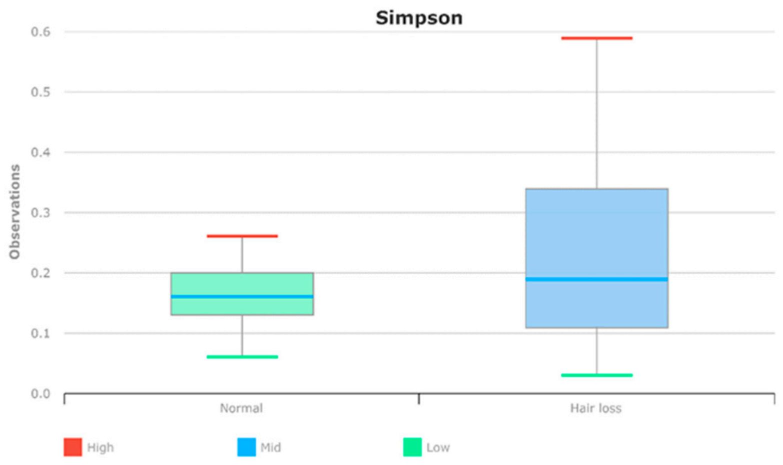

Figure S2. Species equality index from the general public and individuals with hair loss.

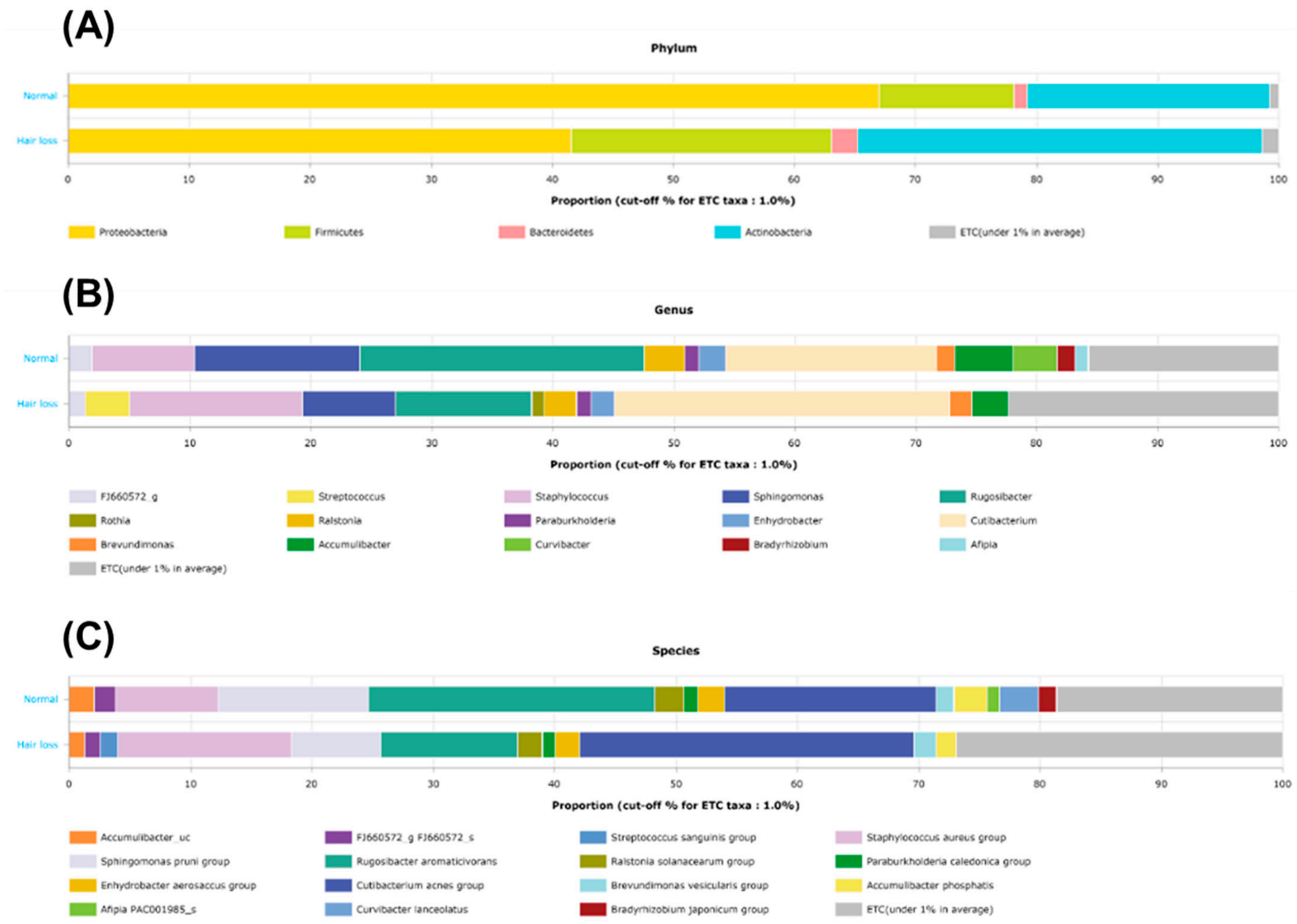

Figure S3. Microbiome strain distribution of the control group and hair loss group at the phylum (A), genus (B), and species (C) levels.

Test substances	Color interference			WST-1 reducibility	Additional control group	
	Water for injection			WST-1 solution		
	OD1	OD2	Mean	Color	**Color**	**WST-1**
Blank	0.003	0.002	0.003			
CICARIA	0.003	0.004	0.004	No color turned	**None**	**None**
**Blank collected data**			**0.001**			

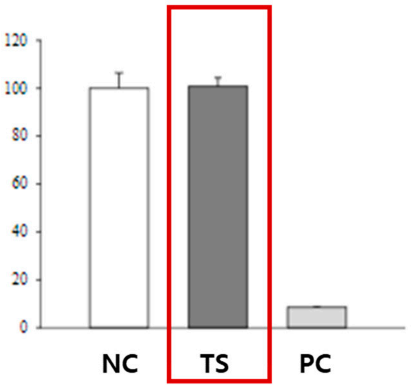

Figure S4. Anti-stimulation test result of Cicaria culture supernatant

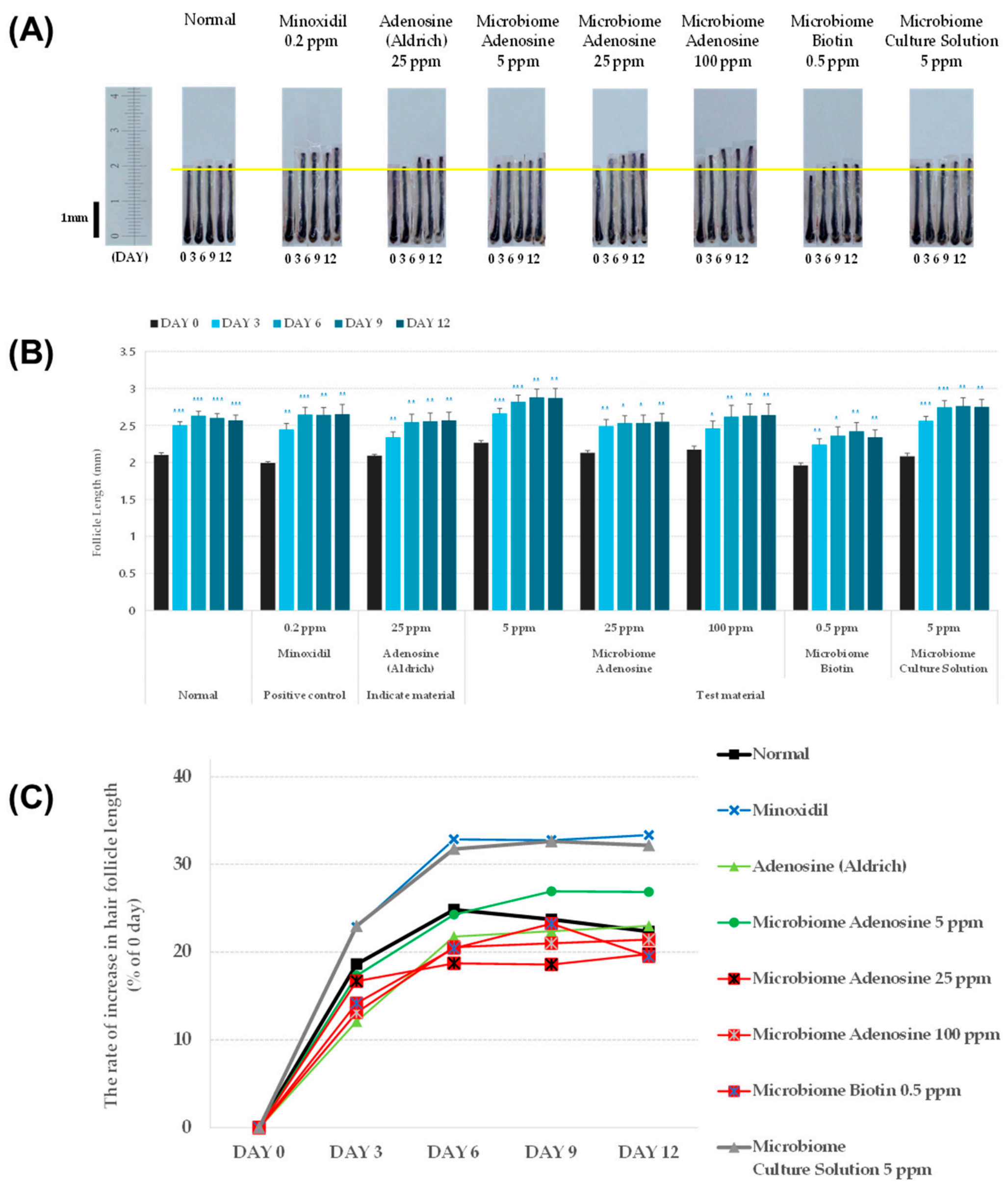

Figure S5. (A) Representative image of changes in hair follicle length by date. Analysis of the hair follicle length (B) and increasing rate (C) (N=9). *p<0.05, **p<0.01, ***p<0.001.
